# CCL5 promotes VEGF-dependent angiogenesis by down-regulating miR-200b through PI3K/Akt signaling pathway in human chondrosarcoma cells

**DOI:** 10.18632/oncotarget.2532

**Published:** 2014-09-26

**Authors:** Guan-Ting Liu, Hsien-Te Chen, Hsi-Kai Tsou, Tzu-Wei Tan, Yi-Chin Fong, Po-Chen Chen, Wei-Hung Yang, Shih-Wei Wang, Jui-Chieh Chen, Chih-Hsin Tang

**Affiliations:** ^1^ Ph.D. Program for Aging, China Medical University, Taichung, Taiwan; ^2^ Department of Orthopedic Surgery, China Medical University Hospital, Taichung, Taiwan; ^3^ School of Chinese Medicine, China Medical University, Taichung, Taiwan; ^4^ Functional Neurosurgery Division, Neurological Institute, Taichung Veterans General Hospital, Taichung, Taiwan; ^5^ Department of Early Childhood Care and Education, Jen-Teh Junior College of Medicine, Nursing and Management, Miaoli County, Taiwan; ^6^ Department of Pharmacology, School of Medicine, China Medical University, Taichung, Taiwan; ^7^ Department of Orthopedic Surgery, Taichung Hospital, Ministry of Health and Welfare, Taichung, Taiwan; ^8^ Department of Nursing, National Taichung University of Science and Technology, Taichung, Taiwan; ^9^ Department of Medicine, Mackay Medical College, New Taipei City, Taiwan; ^10^ Graduate Institute of Basic Medical Science, China Medical University, Taichung, Taiwan; ^11^ Department of Biotechnology, College of Health Science, Asia University, Taichung, Taiwan

**Keywords:** Angiogenesis, CCL5, Chondrosarcoma, miR-200b, VEGF

## Abstract

Chondrosarcoma is the second most common primary malignant bone cancer, with potential for local invasion and distant metastasis. Chemokine CCL5 (formerly RANTES) of the CC-chemokine family plays a crucial role in metastasis. Angiogenesis is essential for the cancer metastasis. However, correlation of CCL5 with vascular endothelial growth factor (VEGF) expression and angiogenesis in human chondrosarcoma is still unknown. CCL5-mediated VEGF expression was assessed by qPCR, ELISA, and Western blotting. CCL5-induced angiogenesis was examined by migration and tube formation in endothelial progenitor cells *in vitro*. CCL5 increased VEGF expression and also promoted chondrosarcoma conditional medium-mediated angiogenesis *in vitro* and *in vivo*. Stimulation of chondrosarcoma with CCL5 augmented PI3K and Akt phosphorylation, while PI3K and Akt inhibitor or siRNA abolished CCL5-induced VEGF expression and angiogenesis. We also demonstrated CCL5 inhibiting miR-200b expression and miR-200b mimic reversing the CCL5-enhanced VEGF expression and angiogenesis. Moreover, in chondrosarcoma patients showed the positive correlation between CCL5 and VEGF; negative correlation between CCL5 and miR-200b. Taken together, results demonstrate CCL5 promoting VEGF-dependent angiogenesis in human chondrosarcoma cells by down-regulating miR-200b through PI3K/Akt signaling pathway.

## INTRODUCTION

Chemokine CCL5 (a.k.a. RANTES) is widely established as an inflammatory chemokine secreted by many cell types: e.g., activated T cell, macrophage, platelet, smooth muscle, endothelial [1]. It is linked with chronic inﬂammatory diseases like bowel disease, rheumatoid arthritis and cancer. Association between its expression and cancer is reported in lung [2], prostate, melanoma, pancreatic, and breast carcinoma [3-5]. Several investigations have been report that CCL5 was detected in samples from patients with breast cancer and the expression levels correlated with disease progression [3, 4]. In osteosarcoma and chondrosarcoma, CCL5 is proven to expedite migration and metastasis [6, 7].

Chondrosarcoma, second most common sarcoma arising in bone malignancy after myeloma and osteosarcoma, is characterized by motility, hyper-vascularity, and migration [8, 9]. Surgical resection remains chief curative therapy, but tumor migration allows poor prognosis [10]. Therefore, better strategies of treatment will ultimately require understanding the molecular mechanisms of the metastasis step of human chondrosarcoma and identifying and specifically targeting the critical signaling effectors [11]. Tumor growth and metastasis depend on cells' ability to induce their own blood supply. Angiogenesis is essential for human cancer progression and development [12]. Vascular endothelial growth factor (VEGF), lead pro-angiogenesis factor, acts directly on endothelial cells to induce proliferation, migration, survival, and finally angiogenesis, facilitating tumor growth. Its expression mediated via PI3K/Akt signaling pathway [13] has been documented. Piccolo *et al*. saw PI3K/Akt signaling pathway activation involved in VEGF-mediated capillary-like tube formation in *breast cancer* [14]. On the other hand, Ping *et al*. found VEGF induction through the PI3K/Akt-dependent signaling pathway in *glioblastoma* [15].

Small (about 22-nucleotides long) non-coding microRNAs (miRNAs) modulate targeted gene expression by either translational repression or mRNA cleavage; one can simultaneously regulate functionally relevant genes, thus reinforcing phenotypic change [16, 17]. They control gene expression by binding to 3′UTRs complementary sequences of target mRNAs [18, 19]. Deregulated expression of miRNAs is cited in human cancer and may affect multiple steps during metastasis [20]. Recent studies report miRNAs involved in angiogenesis and metastatic progression [21, 22]. In addition, miRNAs have been indicated to mediate the progression and metastasis of chondrosarcoma [23, 24]. Earlier we reported CCL5 enhancing chondrosarcoma migration and metastasis via matrix metalloproteinase-3 (MMP-3) up-regulation [6]. VEGF as a potent angiogenic factor is likewise pivotal in angiogenesis and tumor metastasis. We thus hypothesized CCL5 promoting VEGF-dependent angiogenesis in chondrosarcoma. Here we find CCL5 enhancing VEGF expression and subsequently angiogenesis by down-regulating miR-200b through PI3K/Akt signaling pathway.

## RESULTS

### CCL5 promotes VEGF-dependent angiogenesis in human chondrosarcoma

**Figure 1:**
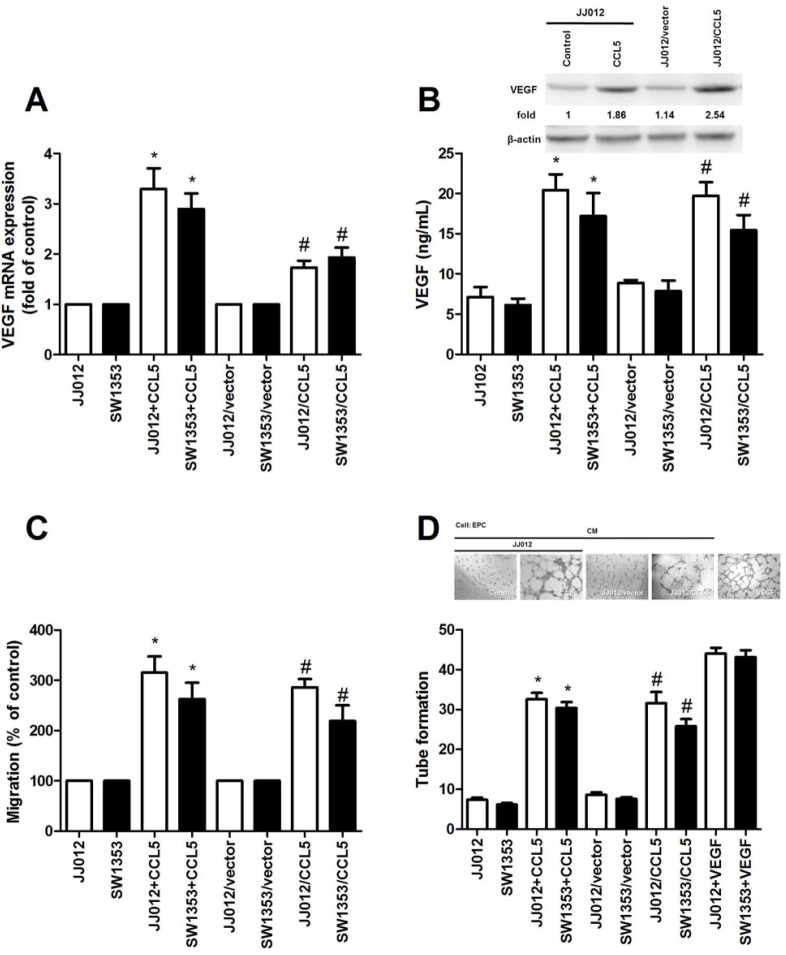
CCL5 boosts VEGF expression, subsequently facilitating angiogenesis in human chondrosarcoma (A&B) JJ012 and SW1353 cells were incubated with CCL5 (100 ng/mL) for 24 h or JJ012/vector, JJ012/CCL5, SW1353/vector and SW1353/CCL5 cells cultured for 24 h, VEGF expression rated by qPCR, ELISA, and western blot (n=5). (C&D) Medium collected as CM was applied to EPCs for 24 h, cell migration and capillary-like structure formation in EPCs examined by Transwell and tube formation assay (n=4-5). Results expressed as mean ± S.E.M. *, *p* < 0.05 compared with control or vector control group; #, *p* < 0.05 compared with CCL5-treated or CCL5- overexpressed group.

CCL5 reportedly promotes migration and metastasis in human chondrosarcoma by up-regulating MMP-3 [6]. VEGF-dependent angiogenesis is plays a key role in metastasis [25]. To evaluate CCL5-induced VEGF expression in chondrosarcoma, we applied human recombinant CCL5 to chondrosarcoma cell lines (JJ012 and SW1353 cells) and assessed VEGF expression. Results showed CCL5 heightening VEGF mRNA expression (Fig. 1A). ELISA assay and western blot analysis found CCL5 inducing production of VEGF (Fig. 1B). Angiogenesis mainly involves endothelial cell proliferation, migration, and tube formation to form new blood vessels [26]. We tested for CCL5-dependent VEGF expression inducing angiogenesis, using EPCs model *in vitro*, to prove conditioned medium (CM) from CCL5-treated chondrosarcoma cells dramatically enhancing migration and tube formation of EPCs (VEGF induces tube formation as positive control; Fig. 1C&D). To elucidate CCL5 effect on VEGF expression and angiogenesis in chondrosarcoma, CCL5 stable transfectant in JJ012 and SW1353 cells were established. After proper selection by antibody, CCL5-overexpressed clone (JJ012/CCL5 and SW1353/CCL5) and vector control (JJ012/vector and SW1353/vector) cells were established. Expression of VEGF increased in CCL5-overexpressed cells (Fig. 1A&B). We also found CM from CCL5-overexpressed cells increasing migration and tube formation of EPCs (Fig. 1C&D); this indicates CCL5-dependent VEGF expression promoting angiogenesis in human chondrosarcoma *in vitro*.

### PI3K and Akt signal pathways involved in CCL5-mediated VEGF up-regulation and angiogenesis in chondrosarcoma

**Figure 2:**
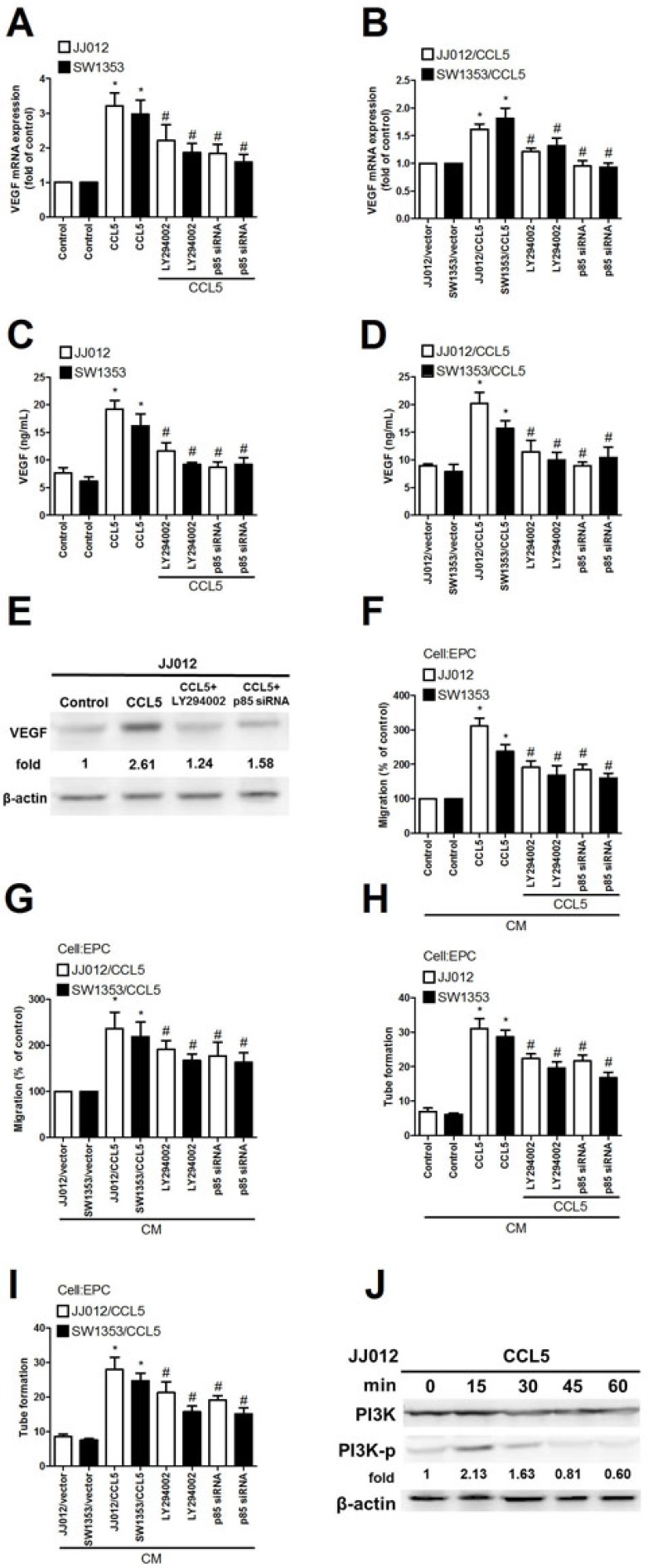
PI3K involved in CCL5-mediated VEGF expression and angiogenesis in human chondrosarcoma (A-E) Cells were pretreated with Ly294002 (10 μM) for 30 min or transfected with p85 siRNA for 24 h, followed by stimulation with CCL5 (100 ng/mL) or vehicle for 24 h, VEGF expression rated by qPCR, ELISA and western blot (n=5). (F-I) Medium was collected as CM and applied to EPCs for 24 h, *in vitro* cell migration and capillary-like structure formation in EPCs examined by Transwell and tube formation assay (n=4-5). (J) JJ012 cells were incubated with CCL5 (100 ng/mL) for indicated time intervals, p-p85 expression examined by western blot (n=3). Results are expressed as mean ± S.E.M. *, *p* < 0.05 compared with control or vector control group; #, *p* < 0.05 compared with CCL5-treated or CCL5- overexpressed group.

The PI3K/Akt signaling pathway plays a lead role in formation of tumor blood vessels [27]. PI3K/Akt signaling also can be activated by growth factors like CCL5 [2]. We hypothesized this pathway's involvement in CCL5-induced VEGF expression and angiogenesis in chondrosarcoma. Pretreatment with PI3K inhibitor Ly294002 markedly inhibited exogenous or overexpressed CCL5-induced VEGF expression as well as EPC migration and tube formation (Fig. 2A-I). To elucidate PI3K effect on CCL5-induced VEGF expression and angiogenesis, p85 siRNA (regulatory subunit of PI3K) was used; transfection with it diminished exogenous or overexpressed CCL5-mediated VEGF expression and angiogenesis (Fig. 2A-I). We tested PI3K phosphorylation after CCL5 treatment; such stimulation of chondrosarcoma cells promoted PI3K phosphorylation time-dependently (Fig. 2J). PI3K is composed of a p110 catalytic subunit and a p85 regulatory subunit [28]. Next, we confirm whether p110 subunit is also involved in CCL5-induced VEGF expression. Indeed, transfection of cells with p110 siRNA diminished CCL5-mediated VEGF expression and angiogenesis (Supplementary Fig. S1).

**Figure 3:**
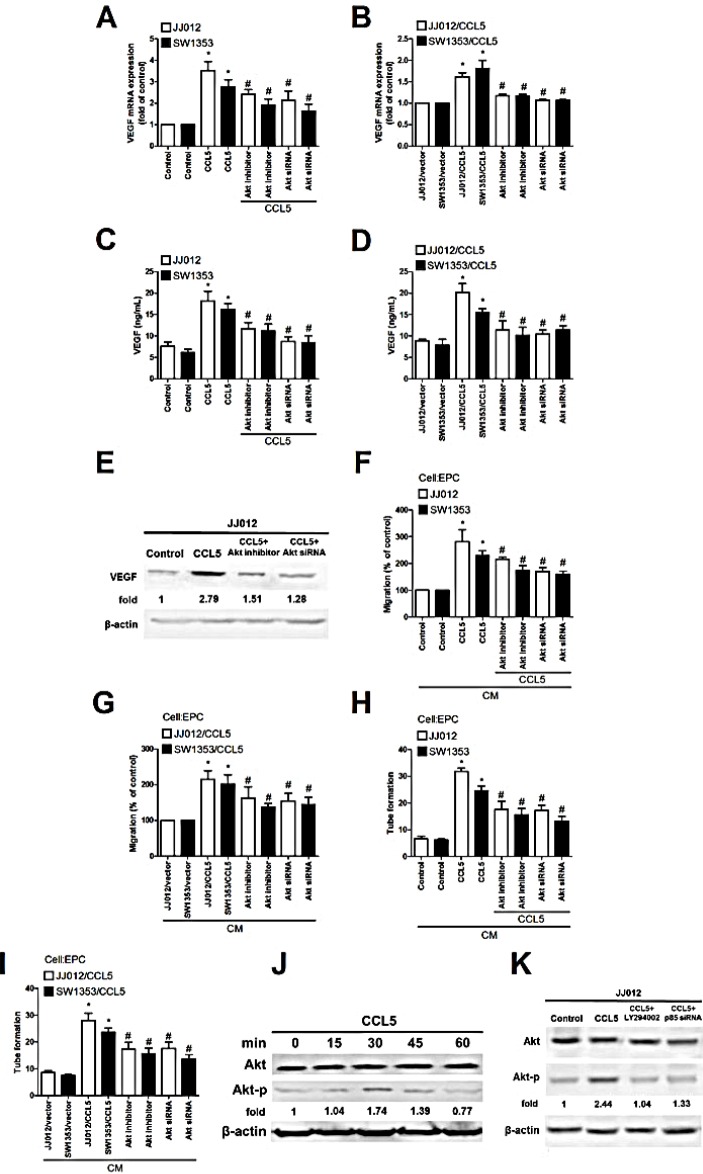
Akt involved in CCL5-mediated VEGF expression and angiogenesis in human chondrosarcoma (A-E) Cells were pretreated with Akt inhibitor (10 μM) for 30 min or transfected with Akt siRNA for 24 h followed by stimulation with CCL5 (100 ng/mL) or vehicle for 24 h, VEGF expression examined by qPCR, ELISA and western blot (n=5). (F-I) Medium was collected as CM and applied to EPCs for 24 h, *in vitro* cell migration and capillary-like structure formation in EPCs examined by Transwell and tube formation assay (n=4-5). (J&K) JJ012 cells were incubated with CCL5 (100 ng/mL) for indicated time intervals or pretreated with Ly294002 for 30 min and transfected with p85 siRNA for 24 h, then stimulated with CCL5 for 30 min. p-Akt expression tested by western blot (n=3). Results are expressed as mean ± S.E.M. *, *p* < 0.05 compared to control or vector control group; #, *p* < 0.05 compared with CCL5-treated or CCL5- overexpressed group.

Previous studies reported CCL5 promoting cancer migration by PI3K-dependent Akt activation [6, 7]. We examined whether Akt is involved in CCL5 triggered VEGF expression and angiogenesis. Pretreatment with Akt inhibitor or siRNA reduced CCL5-mediated VEGF expression, EPCs migration, and tube formation (Fig. 3A-I). We then directly measured Akt phosphorylation in response to CCL5 and found that stimulation of CCL5 significantly increased phosphorylation of Akt (Fig. 3J). On the other hand, CCL5-induced phosphorylation of Akt was diminished by Ly294002 and p85 siRNA (Fig. 3K). Results portend CCL5 acting through PI3K/Akt signal pathway to enhance VEGF expression and angiogenesis in human chondrosarcoma cells. PDK1 is an other upstream molecule of Akt activation [29]. To examine the role of PDK1 in CCL5-mediated Akt activation, the phosphorylation of PDK1 at Thr308 was examined. However, incubation of cells with CCL5 didn't increase PDK1 phosphorylation. Therefore, PDK1 is not involved in CCL5-promoted Akt activation (Supplementary Fig. S2).

### CCL5 promotes VEGF expression and angiogenesis, down-regulating miR-200b

**Figure 4:**
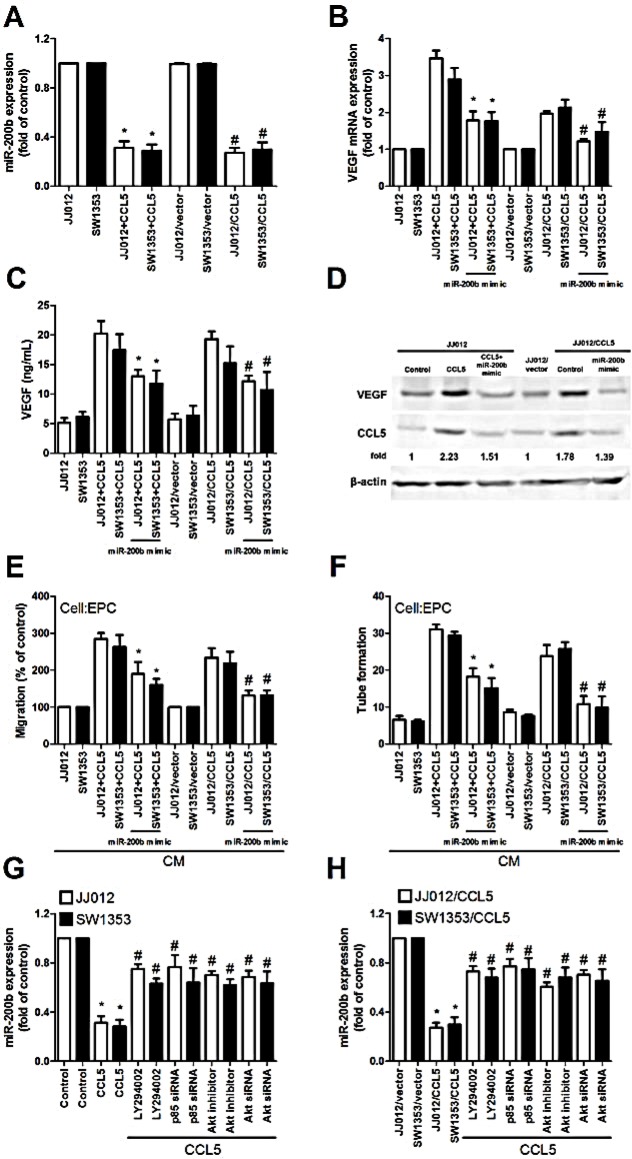
CCL5 promotes VEGF expression and angiogenesis, down-regulating miR-200b (A) Cells were incubated with CCL5 (100 ng/mL) for 24 h or JJ012/vector, JJ012/CCL5, SW1353/vector and SW1353/CCL5 cells cultured for 24 h, miR200b expression examined by qPCR (n=5). (B-D) Cells were transfected with miR-200b mimic for 24 h, followed by stimulation with CCL5 (100 ng/mL) or vehicle for 24 h, VEGF expression rated by qPCR, ELISA and western blot (n=5). (E&F) Medium was collected as CM, then applied to EPCs for 24 h, *in vitro* cell migration and capillary-like structure formation in EPCs examined by Transwell and tube formation assay (n=4-5). (G&H) Cells were pretreated with Ly294002 and Akt inhibitor for 30 min or transfected with p85 and Akt siRNA for 24 h, followed by stimulation with CCL5 (100 ng/mL) or vehicle for 24 h, miR-200b expression examined by qPCR (n=5). Results are expressed as mean ± S.E.M. *, *p* < 0.05 compared with control or vector control group; #, *p* < 0.05 compared with CCL5-treated or CCL5- overexpressed group.

These miRNAs reportedly mediate VEGF expression and angiogenesis in human cancer cells [30, 31]. In addition, miR-200b regulating VEGF-dependent metastasis, tumorigenesis and angiogenesis is documented [32-35]. We hypothesized miR-200b mediating CCL5-promoted VEGF-dependent angiogenesis in human chondrosarcoma. Exogenous or overexpressed CCL5 reduced miR-200b expression (Fig. 4A). To explore miR-200b involvement in CCL5-induced VEGF and angiogenesis, miR-200b mimic was used; transfection with miR-200b mimic diminished CCL5-induced VEGF expression, EPCs migration and tube formation (Fig. 4B-F). On the other hand, PI3K or Akt inhibitor and siRNA reversed CCL5-inhibited miR-200b expression (Fig. 4G&H), indicating CCL5 suppressing miR-200b expression via PI3K/Akt pathway.

**Figure 5:**
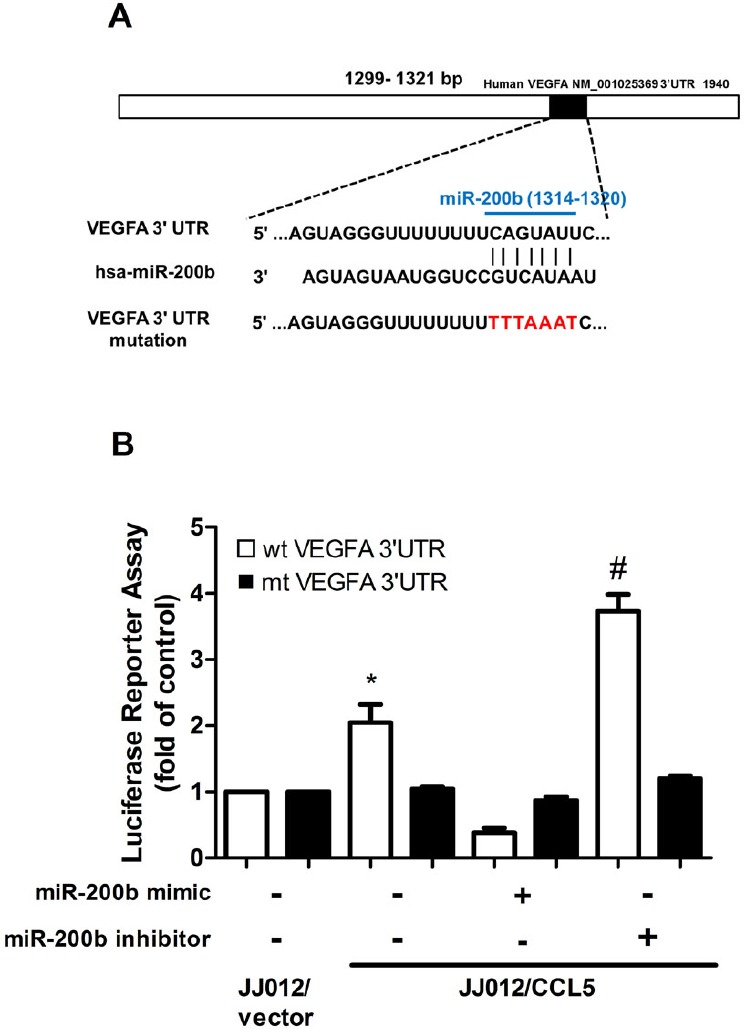
miR-200b directly represses VEGF expression via binding to 3′UTR of human *VEGF-A* (A) Schematic 3′UTR representation of human VEGF-A containing miR-200b binding site. (B) Cells were co-transfected with miR-200b mimic or inhibitor and wt-VEGFA-3′UTR or mt-VEGFA-3′UTR plasmid for 24 h, relative luciferase/renilla activities measured as described in the Methods section. Results are expressed as mean ± S.E.M. *, *p* < 0.05 compared with vector control group; #, *p* < 0.05 compared with CCL5-overexpressed group.

To learn whether miR-200b regulates 3′UTR of VEGF, we constructed luciferase reporter vectors harboring wildtype 3′UTR of VEGF mRNA (wt-VEGFA-3′UTR) and vector containing mismatches in predicted miR-200b binding site (mt-VEGFA-3′UTR) and transfected vectors into JJ012/CCL5 and control cells (Fig. 5A). Cotransfection with miR-200b mimic reduced luciferase activity in wt-VEGFA-3′UTR plasmid but not in mt-VEGFA-3′UTR plasmid (Fig. 5B). Taken together, data indicate miR-200b directly repressing VEGF-A protein expression via binding to 3′UTR of human *VEGF-A* gene.

### CCL5 boosts angiogenesis and tumor growth, down-regulating miR-200b ***in vivo***

**Figure 6:**
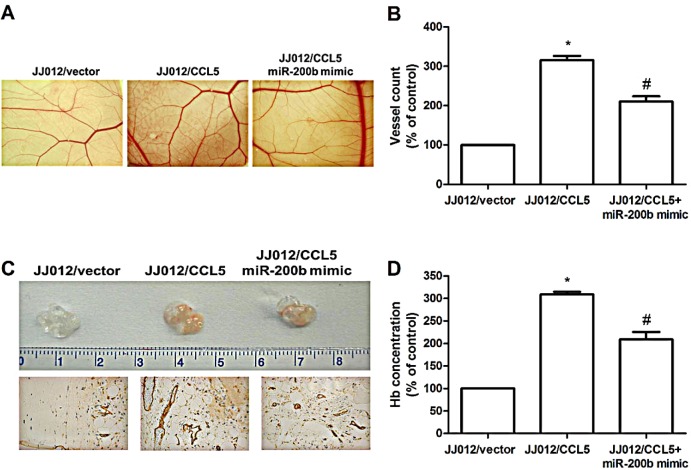
CCL5 promotes angiogenesis by down-regulating miR-200b ***in vivo*** (A&B) Chick embryos were incubated with indicated chondrosarcoma CM for 4 days, then resected, ﬁxed, and photographed by stereomicroscope. (n=5) (C&D) Mice were injected subcutaneously with Matrigel mixed with indicated chondrosarcoma CM for seven days. Plugs excised from mice and photographed were stained with CD31, hemoglobin content quantiﬁed (n=6). Results are expressed as the mean ± S.E.M. *, *p* < 0.05 compared with control; #, *p* < 0.05 compared to CCL5-overexpressed group.

Effect of CCL5 on angiogenesis *in vivo* was rated by CAM assay *in vivo* model. It was clearly noted that CM from JJ012/CCL5 cells increased CAM angiogenesis, and cotransfection with miR-200b mimic blocked CCL5-mediated angiogenesis (Fig. 6A-B). We next performed Matrigel implant assay in mice to further confirm CAM model results: Matrigel mixed with CM from JJ012/CCL5 cells increased microvessel formation (including Matrigel plugs analyzed for CD31 expression and hemoglobin content); miR-200b mimic abolished this effect (Fig. 6C-D). These indicate CCL5 promoting angiogenesis through down-regulation of miR-200b expression *in vivo*.

**Figure 7:**
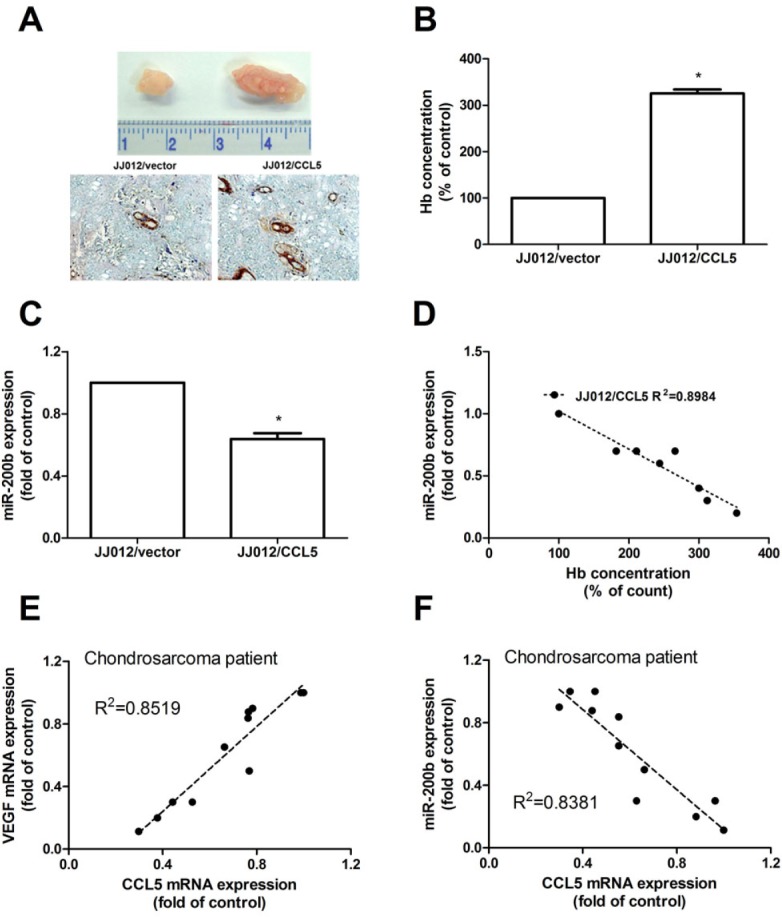
CCL5 increases tumor-associated angiogenesis and tumor growth by down-regulating miR-200b ***in vivo*** Cells were mixed with Matrigel and injected into ﬂank sites of mice sacrificed after six weeks. (A-C) Tumors were photographed with microscope, hemoglobin quantified and examined miR-200b expression (n=6). Correlation between hemoglobin level and miR-200b expression was shown in (D). Correlation between CCL5/VEGF (E) and CCL5/miR-200b (F) in chondrosarcoma cases (n=10), results expressed as mean ± S.E.M. *, *p* < 0.05 compared with control.

We then investigated whether CCL5 could promote tumor angiogenesis *in vivo*. Human chondrosarcoma cells were mixed with Matrigel and injected into flanks of nude mice. Figure 7A shows overexpression of CCL5 promoting tumor growth. We also gauged hemoglobin content and miR-200b expression to find CCL5 increasing chondrosarcoma-induced angiogenesis but reducing miR-200b expression *in vivo* (Fig. 7B-C); hemoglobin content inversely correlated with miR-200b expression (Fig. 7D). We next correlated among miR-200b, VEGF, and CCL5 in chondrosarcoma. Positive correlation between CCL5 and VEGF versus negative between CCL5 and miR-200b (Fig. 7E-F) indicated CCL5 promoting VEGF-dependent angiogenesis and tumor growth by down-regulating miR-200b *in vivo*.

## DISCUSSION

Chondrosarcoma differs from other mesenchymal malignancies, such as Ewing's sarcoma and osteosarcoma, for which the advent of systemic chemotherapy upgraded long-term survival dramatically [36]. Lack of effective adjuvant therapy means poor prognosis for chondrosarcoma. Metastatic potential for conventional chondrosarcoma correlates strongly with histologic grade of a tumor. Due to relatively indolent growth rates of many low- and moderate-grade chondrosarcomas, a mere fifteen percent of patients live over five years after initial diagnosis of metastatic disease [6, 37]. Angiogenesis is a critical step in human cancer metastasis, making it important to develop potential targets for preventing chondrosarcoma angiogenesis. We showed overexpression of CCL5 promoting VEGF expression and increasing angiogenesis *in vitro* and *in vivo.* One mechanism underlying CCL5 increased VEGF production and angiogenesis by down-regulating miR-200b through PI3K and Akt signaling pathway, rendering CCL5 a novel target for chondrosarcoma angiogenesis and metastasis

The newly identified small noncoding miRNAs, a novel class of gene regulators, control gene expression by binding to complementary 3′UTR sequences of target mRNA [18, 19]. The miR-200 family is well known to inhibit epithelial-mesenchymal transition and tumor metastasis [38]; miR-200b is cited as negative regulator of VEGF expression and angiogenesis in lung adenocarcinoma [39], but its effect on human chondrosarcoma is largely unknown. We saw exogenous or overexpression of CCL5 reducing miR-200b expression. Cotransfection with miR-200b mimic abolished CCL5-mediated VEGF expression and angiogenesis. We likewise indicated miR-200b directly repressing VEGF-A protein expression, binding with 3′UTR of human *VEGF-A* gene to negate VEGF-mediated angiogenesis and tumor growth.

PI3K, potential candidate signaling molecule, has shown capacity for regulating VEGF expression [40]. Pretreatment of chondrosarcoma cells with PI3K inhibitor Ly294002 antagonized increase of VEGF production and angiogenesis by CCL5, as confirmed by siRNA against p85 reducing enhancement of VEGF expression and angiogenesis via CCL5 stimulation. One downstream effector of PI3K is serine/threonine kinase Akt [41]. Our study found Akt inhibitor or siRNA reducing CCL5-increased VEGF expression and angiogenesis. In addition, CCL5 promoted Akt phosphorylation, while PI3K inhibitor or siRNA reversed CCL5-enhanced Akt phosphorylation. Therefore, PI3K-dependent Akt pathway play key role in CCL5-mediated VEGF expression and angiogenesis. It has been reported that transcriptional and posttranscriptional regulation play key role in miRNA activation and inhibition [42, 43]. In the current study, cells incubation with PI3K and Akt inhibitor or siRNA reversed CCL5-reduced miR-200b expression indicating CCL5 inhibited miR-200b expression through PI3K/Akt pathway. Whether PI3K/Akt control miR-200b expression through transcriptional or posttranscriptional regulation is needs further examination.

## CONCLUSIONS

**Figure 8:**
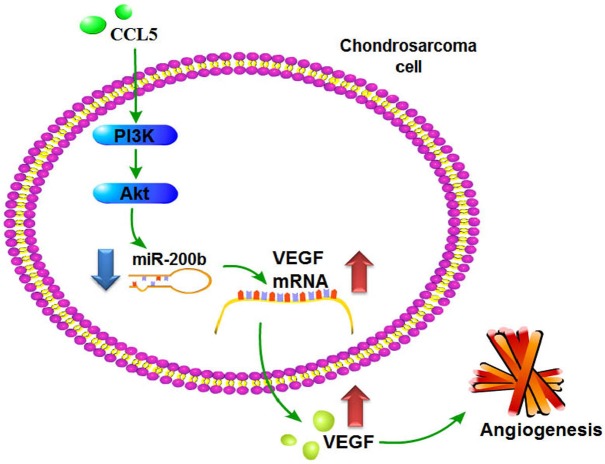
Schema of signaling pathways involved in CCL5-induced VEGF expression and angiogenesis in chondrosarcoma cells CCL5 enhances VEGF-dependent angiogenesis in human chondrosarcoma cells by down-regulating miR-200b through PI3K/Akt signaling pathway.

Metastasis may imply cells from the primary tumor wandering around the body. Most common features are described as invasion, intravasation, and extravasation from the circulatory system, colonization and distant angiogenesis, the latter generally connected with poor prognosis. Anti-angiogenesis has demonstrably suppressed metastasis and benefitted survival in chondrosarcoma patients [44]; anti-angiogenic and -metastatic therapy conceivably can benefit these cases. We observed CCL5 inducing VEGF expression and subsequently promoting angiogenesis and tumor growth through down-regulating miR-200b via PI3K/Akt signaling pathway (Fig. 8). These may lend insight into metastatic mechanisms and effective therapies.

## Materials and Methods

### Materials

Anti-mouse and anti-rabbit IgG-conjugated horseradish peroxidase, rabbit polyclonal antibodies speciﬁc for p-p85, p85, p-Akt, Akt, β-actin and CD31 were purchased from Santa Cruz Biotechnology (Santa Cruz, CA); CCL5 and VEGF antibodies from Abcam (Cambridge, MA); recombinant human VEGF and BX759 from R&D Systems (Minneapolis, MN); recombinant human CCL5 and VEGF ELISA kit from PerpoTech (Rocky Hill, NJ); miR-200b mimic, miR-200b inhibitor, *Lipofectamine 2000, and* Trizol from Life Technologies (Carlsbad, CA). ON-TARGET plus siRNA of p85, Akt (Akt1), and control were purchased from Dharmacon Research (Lafayette, CO); Dulbecco's modiﬁed Eagle's medium (DMEM), α-minimum essential medium (MEM), fetal bovine serum (FBS) and other cell culture reagents from Gibco-BRL Life Technologies (Grand Island, NY); dual-luciferase reporter assay kit from Promega (Madison, WI); PI3K inhibitor (Ly294002), Akt inhibitor (broad Akt1/2/3 inhibitor) and all other chemicals from Sigma-Aldrich (St Louis, MO).

### Cell culture

Human chondrosarcoma cell line (JJ012) was kindly provided by the laboratory of Dr. Sean P. Scully (University of Miami School of Medicine, Miami, FL) and human chondrosarcoma cell line (SW1353) was purchased from the American Type Culture Collection, then cultured in Dulbecco's modified Eagle's medium (DMEM)/α-MEM supplemented with 10% fetal bovine serum and 100 units/ml penicillin/streptomycin at 37°C in humidified chamber in 5% CO_2_.

### Generation of stable cell lines

Cells were seeded on plates and infected with pLKO_AS2.puro-vector or pLKO_AS2.puro-CCL5 by prepared lentivirus. At 24 h after transfection, stable transfectants were selected with puromycin, selection medium thereafter replaced every other day. After two weeks of selection, resistant clones were established.

### Isolation and cultivation of endothelial progenitor cells (EPCs)

Ethical approval was granted by the Institutional Review Board of Mackay Medical College, New Taipei City, Taiwan (reference number: P1000002); informed consent obtained from healthy donors before collection of peripheral blood (80 mL). The peripheral blood mononuclear cells (PBMCs) were fractionated from other blood components by centrifuge on a Ficoll-Paque Plus (Amersham Biosciences, Uppala, Sweden), as per manufacturer's instructions. CD34-positive progenitor cells were obtained from the isolated PBMCs using a CD34 MicroBead kit and a MACS Cell Separation System (Miltenyi Biotec, Bergisch Gladbach, Germany). Maintenance and characterization of CD34-positive EPCs were performed as described previously [45, 46]. Briefly, human CD34-positive EPCs were maintained and propagated in MV2 complete medium consisting of MV2 basal medium and growth supplement (PromoCell, Heidelberg, Germany), with 20% chemically defined FBS (HyClone, Logan, UT). Cells were seeded onto 1% gelatin-coated plastic ware and cultured in humidified air containing 5% CO_2_ at 37°C for further treatment.

### Preparation of conditioned medium (CM)

In the series of experiments, cells were treated with CCL5 alone for 24 h, or pretreated with pharmacological inhibitors including Ly294002, p85 siRNA, Akt inhibitor or Akt siRNA followed by stimulation with CCL5 for 24 h. After treatment, cells were washed and changed to serum-free medium. CM was then collected 2 days after the change of medium and stored at −80°C until use.

### ELISA assay

Cells (2×10^4^) were cultured in 24-well culture plates and incubated in a humidified incubator at 37°C for 24 h. After pretreatment with Ly294002, p85 siRNA, Akt inhibitor or Akt siRNA, and followed by stimulation with CCL5 for 24 h, the medium was removed and stored at −80°C until assay. VEGF in the medium was assayed using the VEGF enzyme immunoassay kits Peprotech (Offenbach, Germany), according to the procedure described by the manufacturer.

### Western blot analysis

Cells were collected and lysed in cold RIPA buffer with protein inhibitors, proteins resolved on SDS-PAGE and transferred to Immobilon polyvinyldifluoride (PVDF) membranes. Blots were blocked with 4% BSA for 1 h at room temperature, then probed with rabbit anti-human antibodies against CCL5 or VEGF (1:1000) for 1 h at room temperature. After three washes, blots were subsequently incubated with a donkey anti-rabbit peroxidase-conjugated secondary antibody (1:1000) for 1 h at room temperature and visualized by enhanced chemiluminescence, using Imagequant LAS 4000 (GE Healthcare, Pewaukee, WI) [47].

### Quantitative real-time PCR (qPCR) of mRNA and miRNA

This analysis was conducted with Taqman^®^ one-step PCR Master Mix (Applied Biosystems, CA), 100 ng of total cDNA added per 25 μl reaction with sequence-specific primers and Taqman^®^ probes. Sequences for all target gene primers and probes (GAPDH as internal control) were purchased commercially (Applied Biosystems, CA). qPCR assays were conducted in triplicate by StepOnePlus sequence detection system. Cycling conditions: 10-min polymerase activation at 95°C followed by 40 cycles at 95°C for 15 s, 60°C for 60 s. Threshold was set above non-template control background and within linear phase of target gene amplification to calculate cycle number at which transcript was detected (denoted C_T_) [48].

For miRNA assay, cDNA was synthesized from total RNA (100 ng) by TaqMan MicroRNA Reverse Transcription Kit (Applied Biosystems), reactions incubated first at 16°C for 30 min and at 42°C for 30 min, followed by inactivation at 85°C for 5 min, then incubated in a 96-well plate at 50 °C for 2 min, 95°C for 10 min, followed by 30 cycles of 95°C for 15 s and 60°C for 60 s, by StepOnePlus sequence detection system. Relative gene expression quantification used endogenous control gene (U6). Threshold cycle (CT) was defined as fractional cycle number at which fluorescence passed fixed threshold, relative expression calculated by comparative CT method.

### Plasmid constructs

The 3′UTR-luciferase reporter constructs containing 3′UTR regions of VEGF with wild type and mutant binding sites of miR-200b were amplified by PCR method, cDNAs obtained from H293T cells. PCR products were cloned into *pmirGLO* reporter vector (Promega) between *Pme*I and *Xho*I sites, instantly downstream of the luciferase gene. Mutant 3′UTR were constructed by introducing seven mismatched mutations into putative seed regions of VEGF, with all constructs containing 3′UTR inserts sequenced and verified.

### Luciferase reporter assay

Cells were seeded on 6-well plates, then were transiently transfected with VEGF 3′UTR luciferase plasmids by *Lipofectamine 2000,* as per manufacturer's instructions. Cells collected were lysed with reporter lysis buffer 24 h after transfection, luciferase and renilla activities in cellular extracts were determined by dual-luciferase^®^ reporter assay system. Relative luciferase activity was calculated by ratio of luciferase/renilla activity, and normalized to that of control cells.

### Migration assay

The migration assay was performed using Transwell 24-well dishes with a pore size of 8 μm (Costar, NY, USA). Approximately 1×10^4^ EPCs in 200 μl of DMEM serum-free medium were placed in the upper chamber, and 300 μl of medium containing 50% serum-free DMEM and 50% CM was placed in the lower chamber. The cells were incubated for 24 h at 37°C in 5% CO_2_, then fixed in methanol for 15 min and stained with 0.05% crystal violet in PBS for 15 min. Cells on the upper side of the filters were removed with cotton-tipped swabs and the filters were washed with PBS. Cells on the underside of the filters were examined and counted under a microscope. Each clone was plated in triplicate in each experiment and each experiment was repeated at least three times.

### Tube formation assay

Matrigel (BD Biosciences; Bedford, MA) was dissolved at 4°C overnight, and 48-well plates were prepared with 150 μL Matrigel in each well after coating and incubating at 37°C for 30 min. EPCs (5 × 10^4^ cells) were given 100 μL cultured media which included 50% MV2 complete medium and 50% osteosarcoma cell conditioned medium. After 16 h of incubation at 37°C, EPC tube formation was imaged with the inverted phase contrast microscope. Tube branches and total tube length were calculated using MacBiophotonics Image J software.

### Hemoglobin assay

All the sponges (Matrigel plugs or tumors) were processed for measurement of angiogenesis. Briefly, the concentrations of hemoglobin in the vessels that had invaded the Matrigel or tumor were measured with Drabkin's reagent (Sigma-Aldrich, St. Louis, MO) according to manufacturer's instructions. Tissues were then homogenized in 1 ml of RIPA lysis buffer, centrifuged at 1000 rpm, and then 20 μl of the supernatant was added to 100 μl of Darkin's solution. The mixture was allowed to stand for 30 min at room temperature, and then readings were taken at 540 nm using a spectrophotometer. The results are expressed in milligrams per milliliter.

### Chorioallantoic membranes (CAM) assay

Fertilized chicken eggs were incubated at 37°C and 80 % humidiﬁed atmosphere. On Day 8, chondrosarcoma cell CM was deposited in the center of the chorioallantoic. Angiogenesis responses were analyzed four days after implantation. Number of blood vessels as index of angiogenesis was counted by branches of blood vessels. At least 10 viable embryos were tested for each treatment, all animal experiments conducted in accordance with protocol approved by the China Medical University (Taichung, Taiwan) Institutional Animal Care and Use Committees.

### Matrigel plug assay

JJ012/vector and JJ012/CCL5 cells were transfected with miRNA control or miR-200b mimic for 24 h. CM was then collected after 2 days. Mice were subcutaneously injected with 0.2 ml Matrigel containing 0.1 ml chondrosarcoma CM. Matrigel pellets were harvested 7 days after implantation, ﬁxed with 4% paraformaldehyde/PBS, and embedded in parafﬁn. Samples were processed later for CD31 staining; blood vessel formation of samples was also quantified by the Drabkin method with Drabkin's reagent kit.

### Tumor xenograft ***in vivo*** study

Experimental cells were implanted into 10 nude mice by subcutaneous injection: 1 × 10^6^ chondrosarcoma cells resuspended in 0.1 ml of serum-free DMEM/α-MEM and injected into right flanks of thirty four-week-old male nude mice. After six weeks, mice were sacrificed and tumors excised for hemoglobin assay. Mice were observed daily and body weights monitored for toxicity. Tumor volume and weight were also measured during this experiment.

### Statistical analysis

Data signify mean ± standard error of mean (SEM); one-way ANOVA (analysis of variance) with Bonferroni multiple comparison ascertained intergroup differences. Student's *t*-test plotted significance between groups, *p<*0.05 considered signiﬁcant.

## SUPPLEMENTAL MATERIAL FIGURES


