# Supplementary material for: CCL5 promotes VEGF-dependent angiogenesis by down-regulating miR-200b through PI3K/Akt signaling pathway in human chondrosarcoma cells
Source: Oncotarget. 2014 Sep 26;5(21):10718–31. doi: 10.18632/oncotarget.2532 (PMC4279405; doi:10.18632/oncotarget.2532)
Supplement: Supplementary file 1 [file oncotarget-05-10718-s001.pdf]

CCL5 promotes VEGF-dependent angiogenesis by down-regulating miR-200b through PI3K/Akt signaling pathway in human chondrosarcoma cells

Supplementary Material

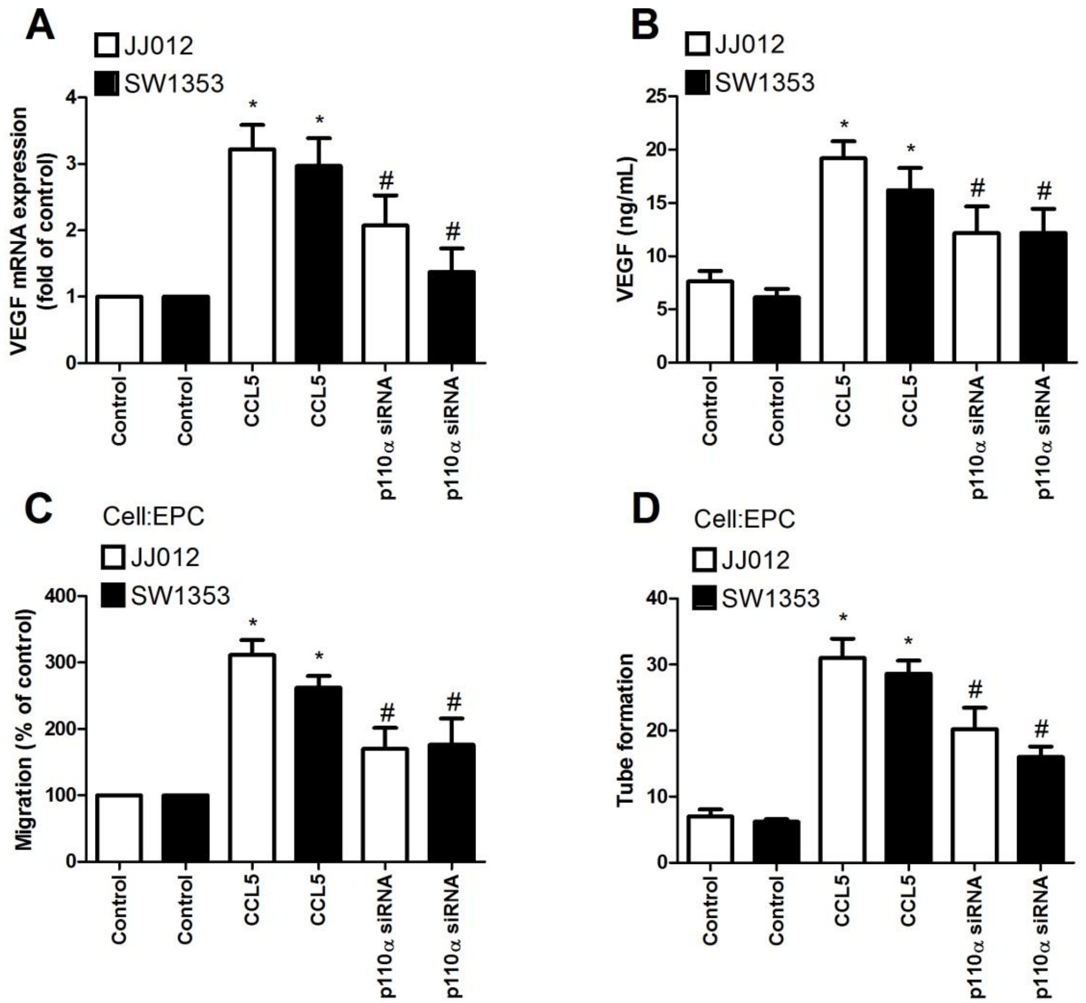

Fig. S1: p110 subunit involved in CCL5-mediated VEGF expression and angiogenesis in human chondrosarcoma. (A&B) Cells were transfected with p110α siRNA for 24 h followed by stimulation with CCL5 (100 ng/mL) for 24 h, VEGF expression rated qPCR and ELISA. (C&D) Medium was collected as CM and applied to EPCs for 24 h, *in vitro* cell migration and capillary-like structure formation in EPCs examined by Transwell and tube formation assay. Results are expressed as mean ± S.E.M. *, $p < 0.05$ compared with control group; #, $p < 0.05$ compared with CCL5-treated group.

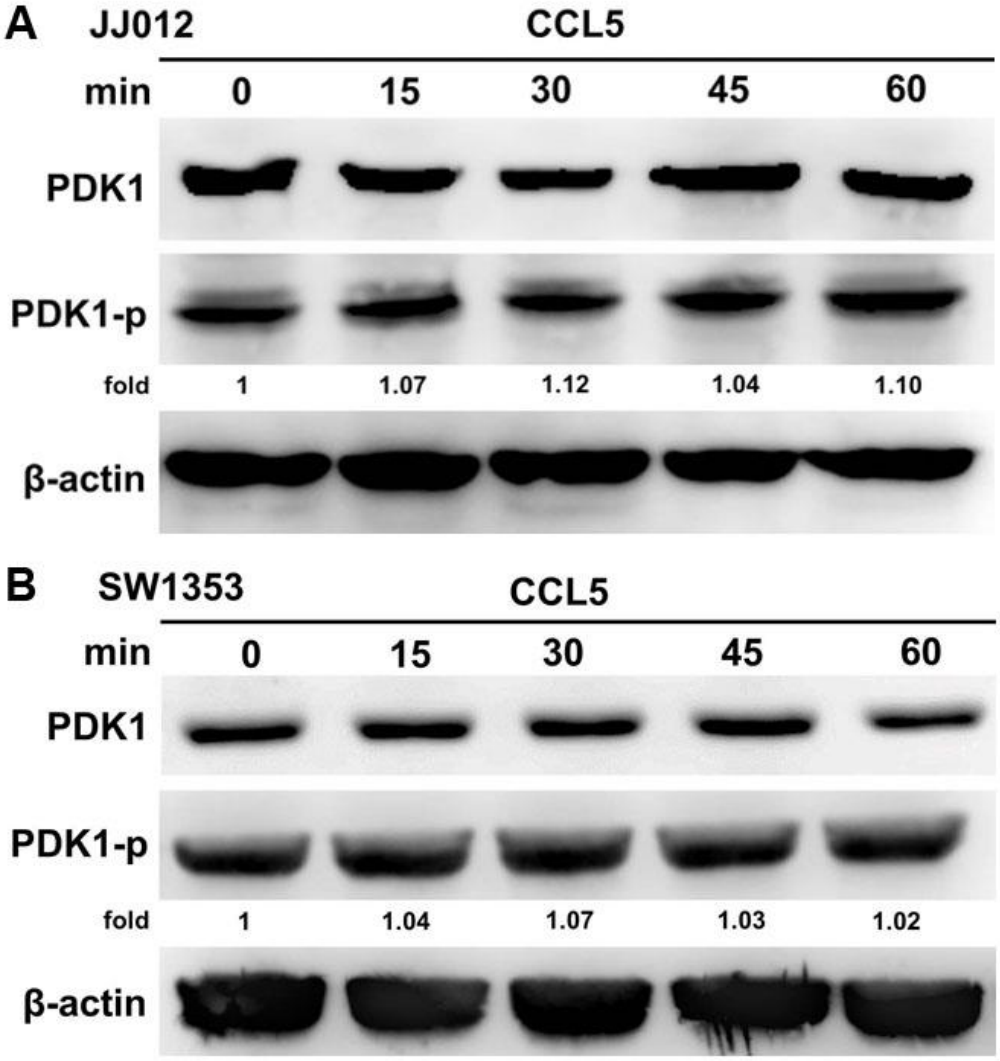

Fig. S2: CCL5 did not increase PDK1 phosphorylation in human chondrosarcoma cells. Cells were incubated with CCL5 (100 ng/mL) for indicated time intervals, the p-PDK1 expression examined by western blot. Results of three independent experiments performed are expressed.
